# Chronic cannabis use in people with bipolar disorder is associated with comparable decision-making and functional outcome to healthy participants

**DOI:** 10.1038/s41398-025-03718-4

**Published:** 2025-11-27

**Authors:** Alannah Miranda, Benjamin Z. Roberts, Breanna M. Holloway, Elizabeth Peek, Holden Rosberg, Samantha M. Ayoub, Daniele Piomelli, Kwang-Mook Jung, Samuel A. Barnes, Steven Rossi, Mark A. Geyer, William Perry, Arpi Minassian, Jared W. Young

**Affiliations:** 1 Department of Psychiatry, University of California San Diego, San Diego, US; 2 Department of Anatomy and Neurobiology, University of California, Irvine, US; 3 Department of Research, VA San Diego Healthcare System, San Diego, US

**Keywords:** Bipolar disorder, Clinical pharmacology

## Abstract

Impaired decision-making is often seen in people with bipolar disorder (BD), even those undergoing treatment. Targeted therapeutics are therefore needed. People with BD report that cannabis use (CU) attenuates such cognitive and behavioral symptoms. We hypothesized that 1) people with BD who do not use cannabis would exhibit poor decision-making and functional capacity relative to healthy comparison (HC) participants and 2) CU in people with BD would be associated with decision-making and functional capacity comparable to that of HC participants who do not use cannabis. HC and BD participants that either reported regular (≥4x/weekly) CU or no-CU were recruited (n = 87). Participants were tested on decision-making and functional capacity using the Iowa Gambling Task and UCSD Performance-based skills assessment (UPSA-2), respectively. CU was associated with impaired decision-making in healthy participants while CU in participants with BD was associated with better decision-making than their non-using counterparts and equivalent to decision-making in non-CU HC participants. Additionally, CU in people with BD was associated with UPSA-2 scores comparable to non-CU HC participants. Studies are needed to determine whether cannabinoid-related treatments improve such decision-making and function in people with BD.

## Introduction

People with bipolar disorder (BD) often struggle with deficits in goal-directed behaviors such as decision-making and inhibitory control. These behavioral and cognitive deficits can often lead to disruptions in social, occupational, and family life, as well as poor health outcomes [1]. Identifying mechanisms underlying these deficits may enable the development of targeted treatments, thereby improving the lives of people with BD. Self-medication hypotheses of drug use in people with BD (i.e., the premise that these individuals use certain drugs to manage symptoms), reveal potential avenues of research. For example, cannabis use (CU) is exceptionally prevalent among people with BD, with over 70% reporting a lifetime history of regular CU versus 26% of the general population [2]. Prevalence of cannabis use continues to increase as its legalization and availability continues to grow. People with BD consistently report using cannabis to ameliorate cognitive and behavioral symptoms such as racing thoughts and hyperactivity [3–5]. Not surprisingly, cannabis is the most commonly used drug among people with BD [6].

Regular CU is often associated with cognitive deficits and risk-taking behaviors however [7, 8], calling into question its utility for self-medication and raising concerns for potential exacerbation of cognitive deficits in people with BD. This contrast in potential effects in people with BD was highlighted by a recent review by our group [9], which identified two studies reporting associations between CU and improved cognitive performance [10, 11], one study reporting an association with poorer cognitive performance [12], and three studies reporting no associations [13–15]. The markedly different criteria for CU and BD status, as well as the cognitive domains tested, may have contributed to these equivocal results. Nevertheless, considering the equivocal results of our review and continued patient reports of self-medication, more research is needed to better understand the relationship between CU and cognitive functions in people with BD. This need is additionally underscored by the increase in therapeutic CU concomitant with cannabis legalization across the United States [16]. An overall increase in CU [16], combined with its potential for improving cognition in BD, highlights the importance of research investigating cognitive domains in this population.

Decision-making is among the most critically affected cognitive domains in BD, with deficits being evident at all stages of the disorder [17]. Deficits in decision-making likely contribute to increased engagement in risky and impulsive behaviors characteristic of people with BD. While decision-making is a broad cognitive domain impacting everyday functioning, numerous tasks exist that can assess such functions in laboratory settings such as the Iowa Gambling Task (IGT), which has real-world significance [18]. While many clinical populations exhibit poor performance in the IGT, their performance is driven by different decision-making strategies, e.g., high-risk high-reward preference in people with BD versus elevated punish-sensitivity in people with depression [19]. A major advantage of the IGT is that it can be translated across species, thus enabling investigation of mechanisms underlying elevated risk preference in rodent models relevant to BD [20–22]. We previously demonstrated that mice with reduced expression of the dopamine transporter (DAT) exhibit deficient decision-making in the IGT as seen in people with BD, i.e., elevated overall risk preference driven by more frequent choice of risky options following receipt of small, high-probability rewards (elevated safe win-stay) [18]. Further, reduced DAT function in mice results in behavior consistent with people with BD including hyper-exploration [23, 24], inattention [25], and poor decision-making in the IGT [20, 26, 27]. Reduced DAT expression (from positron emission studies) was seen in unmedicated people with BD euthymia [28] and mania [29], and may arise from polymorphisms in the DAT gene [24] associated with BD [30, 31]. These mechanistic and behavioral links enable future translational and treatment studies using animals.

Reduced DAT expression drives hyperdopaminergia [32] which may play a role in BD [27–29, 33–35]. The primary constituent of cannabis, delta-9-tetrahydrocannabinol (THC), affects dopamine through indirect DAT interactions, particularly in brain regions that control cognitive functions affected in BD [36]. Acute THC administration activates the endocannabinoid (eCB) system to promote dopamine release [37]. On the other hand, chronic THC reduces dopamine transmission as revealed by changes in dopamine D2/3 receptor signaling in non-human primates [38] and rodents [39]. Dopaminergic signaling plays a critical role in cognitive function, including decision-making; thus, chronic CU may exert a unique effect on aberrant dopaminergic signaling in BD, potentially resulting in the observed downstream changes in cognitive function and behavior.

Here, we sought to determine whether chronic CU was differentially associated with decision-making in people with BD versus healthy comparison (HC) participants. First, we investigated the association between CU and cognitive function in BD by assessing 4 groups: HC, HC + CU, BD, and BD + CU on the IGT. Next, to better understand the effects of CU on real-world functional behavior, participants were also tested in the UCSD Performance-Based Skills Assessment (UPSA-2), a role-play test designed to evaluate a person’s functional capacity in selected areas. We hypothesized that people with BD who do not use cannabis would exhibit poorer decision-making and functional capacity compared to non-CU HC participants. In contrast, we hypothesized that CU in BD would be associated with decision-making and functional capacity comparable to non-CU HC participants.

## Patients and methods

### Participants

87 participants (18–50 years old) were recruited from the local area (San Diego, CA) via social media campaigns, online advertisements, and flyers posted in local coffee shops, libraries, bus stops, and community centers. 50 were healthy comparison (HC) participants who had never met DSM-5 criteria for any Axis I psychiatric disorder and the remaining 37 participants were previously diagnosed for any type of BD (e.g., BD I, BD II, cyclothymic, etc.). Diagnoses were confirmed through assessment by trained research staff using the SCID RV/NP (Version 1.0.0). The SCID RV was used to confirm BD diagnosis in our clinical cohort, whereas the NP version was used to assess the healthy comparison cohort to ensure the absence of any exclusionary conditions. Self-report data were collected on current medication use; however, data was limited on other forms of BD treatment (e.g., previous therapy and self-help group attendance, etc.). BD participants were excluded if they reported clinically severe mood symptoms at the time of testing (Young Mania Rating Score >20 [40] and Hamilton Depression Scale> 23 [41]). Participants were also excluded for: (1) current alcohol or substance use disorder (excluding mild, moderate or severe cannabis use disorder for CU groups); (2) a history of neurological conditions, head trauma, or seizures; (3) treatment with electroconvulsive therapy; (4) stroke or myocardial infarction; (5) a positive urine toxicology result for THC (non-CU groups only), other illicit drugs or non-prescribed medications (i.e., cocaine, amphetamine, methamphetamine, methadone, tricyclic antidepressants, opiates, phencyclidine, barbiturates, and benzodiazepines) assessed using a multi-drug screen urine dipstick test; (6) active suicidality (assessed by the SCID and symptom ratings). All participants provided written informed consent to the current protocol approved by the UCSD Institutional Review Board known as the Human Research Protections Program. Both BD and HC participants were recruited based on two groupings: no CU (less than 5x lifetime use and no use in the past 90 days) or + CU (4x/week or more for the past 90 days [42–45]). The four groups (HC, BD, HC + CU, BD + CU) were matched for gender, education, and ethnicity, but age differed significantly between no CU and + CU participants (Table 1). Presence of any current alcohol use and estimated drinks per week (mean = 1.8, SD = 2.5, range = 0–10) was also reported by all participants. Current BD-related medications were self-reported; BD and BD + CU participants were matched on relative proportions reporting medication use in each medication category.

**Table 1:** Demographic information for comparison groups.

	A	B	C	D	
	HC (n = 24)	HC + CU (n = 26)	BD (n = 12)	BD + CU (n = 25)	Group differences
**Age**	34.4 (9.9)	30.4 (8.1)	39.5 (7.2)	28.8 (8.3)	C > D,B KW = 12.8
**Education (years)**	15.7 (2.8)	14.6 (2.1)	14.6 (2.1)	14.0 (2.5)	ns; KW = 5.9
**Gender**					
Man	13	11	5	9	ns; χ^2^ = 8.3
Woman	11	13	6	14	
Non-binary	0	0	1	2	
Trans (Female to Male)	0	2	0	0	
**Race/Ethnicity**					
White	16	16	6	10	ns; χ^2^ = 10.9
Black	2	2	1	3	
Hispanic	3	4	2	9	
Asian	3	2	2	1	
Additional Groups	0	2	1	2	
**Current Alcohol use (% Yes)**	76.2%	88.5%	54.4%	52.2%	B > C,D χ^2^ = 9.4
**# Alcohol drinks/week**	0.8 (1.0)	2.8 (2.7)	1.6 (3.2)	1.6 (2.5)	B > A,C,D KW = 9.9
**Bipolar Type**					
BD I			9	21	ns; χ^2^ = 2.2
BDII			1	4	
Not otherwise specified			2	0	
Current Medication*					
Lithium	0	0	2	4	ns; χ^2^ = 0.0
Valproic Acid	0	0	1	1	ns; χ^2^ = 2.3
Other mood stabilizers	0	0	2	8	ns; χ^2^ = 2.8
Antipsychotic	0	0	7	6	D > C; χ^2^ = 6.2
Antidepressant	1	0	3	7	ns; χ^2^ = 0.1
Benzodiazepine + Hypnotics	0	0	1	2	ns; χ^2^ = 0.3
Stimulant	0	1	0	3	NA
Other anti-anxiety	0	0	3	6	ns; χ^2^ = 0.1
Opioids	0	1	0	1	NA
**Young Mania Rating Score**	2.8 (2.3)	4.0 (3.4)	6.6 (4.5)	5.7 (3.2)	C,D > A KW = 13.1
**Hamilton Depression Scale**	3.9 (2.7)	5.6 (3.8)	9.3 (5.4)	8.0 (4.7)	C,D > B,A KW = 15.8

Healthy comparison participants (HC), participants with bipolar disorder (BD), non-cannabis users and chronic cannabis users ( + CU). Data are presented as counts or means (standard deviation).

*Statistics for current medications are in reference to group differences between BD and BD + CU. Other mood stabilizers include anti-convulsant medication or gabapentin.

### Iowa gambling task

Participants were administered the Iowa Gambling Task (IGT 18]), a computerized decision-making task in which participants are instructed to select from 4 decks of cards (A, B, C, D) that yield hypothetical monetary rewards of various amounts at various levels of risk. After selecting a card, a participant either may win or lose a theoretical amount of money. Decks A and B (risky choices) contain both large amounts of monetary gains, but also large losses compared to Decks C and D (safe choices) which contain smaller amounts of monetary gains but also smaller losses, making decks C and D “lower risk” and more advantageous over time. The primary outcome measure was the Total Net Difference score, calculated by subtracting the total number of risky choices from the total number of safe choices. The secondary IGT outcomes were defined and calculated as follows:

Safe win-stay ratio (SWS): probability of choosing an advantageous option after being rewarded by an advantageous choice; {# safe choices after safe rewards/# safe rewards}

Safe lose-shift ratio (SLS): probability of choosing a disadvantageous option after being punished by an advantageous choice; {# risky choices after safe punishments/# safe punishments}

Risky win-stay ratio (RWS): probability of choosing a disadvantageous option after being rewarded by a disadvantageous choice; {# risky choices after risky rewards/# risky rewards}

Risky lose-shift ratio: (RLS): probability of choosing an advantageous option after being punished by a disadvantageous choice; {# safe choices after risky punishments/# risky punishments}.

### UCSD performance-based skills assessment (UPSA-2)

Participants were administered the UPSA-2 which uses role-play situations in six different domains (comprehension/planning, finance, communication, transportation, household management, medication management) to evaluate functioning and neuropsychological deficits [46]. Performance is scored based on accuracy and completeness of participant responses within each situation. Trained research staff scored participant performance using a standardized scoring sheet. Each domain score ranges from 0–20 points with higher scores reflecting better performance. UPSA scoring was typically completed by research staff immediately after the experimental visit, as such they were not blinded to participant group status. UPSA scoring uses clearly defined scoring criteria (i.e., participants are marked as correct or incorrect) to minimize the potential for scoring bias.

### Cannabis use survey

Participants were asked about their past-year CU. Survey questions included purposes of CU (i.e., recreational, medicinal, or both) and frequency of CU. These data were based on participant recollection, as such detailed data on cannabinoid content or potency (e.g., relative ratios of THC:CBD, or percent THC/CBD) are not available. Participants reported engaging in several modes of administration including inhalation (smoking, vaping) and consumption (edibles). Participants reported using products containing primarily THC or combinations of THC and CBD in addition to other constituents and no participants reported use of products containing primarily CBD. Cannabis use survey data is presented in **Supplemental Data 2**. For CU frequency, participants were asked to indicate number of CU times per day in an average week (e.g., 1 time 7 days/week, 1 time 3 days/week, etc.). Average weekly CU for each participant was calculated using these responses (# of times per day × days per week). Participants were stratified into the following CU frequency groups: no CU (0x/week), moderate CU (4–24x/week) and heavy CU (25x + /week) [46, 47] based on the distribution of CU frequency within this cohort (mean weekly CU frequency = 23.6, SEM = 2.8). Although our ranges are broad and higher than those used in other studies, they are representative of the higher prevalence of cannabis use at the study location and for this participant population. A similar classification has also been used in other published studies [47, 48].

### Statistical analyses

Assumptions for equal variances (Levene’s or Box’s test of equality) and normality (Shapiro-Wilks test) were tested for demographic differences, IGT outcomes, UPSA scores; variance and normality were tested across the entire sample and within each group. Potential outliers were assessed using boxplots and Tukey’s method. Participants with missing cognitive data were excluded from analyses. Kruskal-Wallis tests with follow up pairwise Mann-Whitney U tests, or Chi-square tests were used to determine demographic or CU pattern differences between BD and/or CU groups. Chi-square tests were used to assess differences in current medications used was assessed between BD and BD + CU participants. Clinical and demographic covariates that differed between groups and/or were correlated with outcome variables (i.e., age and mania symptoms) were considered as covariates. Sensitivity analyses were conducted to determine the effects of these factors on outcome variables; the results remained consistent therefore we did not include covariates for these analyses.

IGT Net Difference score and IGT lose-shift ratios were normally distributed, as such these variables were analyzed using a 2 × 2 ANOVA with BD and CU status as between-subjects factors. Cohen’s d or η ^2^ effect sizes were calculated for main effects and interactions. Planned comparison tests t-tests were conducted between the HC group and the other groups, as well as within the BD group by CU status. Given our a priori hypotheses, the α level was set at 0.05.

IGT win-stay ratios and UPSA scores were not normally distributed, as such these variables were analyzed using non-parametric tests. IGT win-stay ratios were analyzed using Kruskal-Wallis tests and planned pairwise Mann-Whitney U tests to assess group differences between HC group and the other groups, as well as within the BD group by CU status. Spearman’s rho correlation coefficients (r_s_) were used to test for correlations between UPSA and IGT scores. UPSA scores were analyzed using the Kruskal-Wallis test and planned pairwise Mann-Whitney U tests, as described above.

Supplemental analyses were then carried out to explore the effect of CU frequency on IGT performance. 2 × 3 ANOVAs were conducted on IGT outcome variables using BD and CU frequency groups (no CU, moderate CU and heavy CU) as between-subjects factors (Supplemental Data). Secondary IGT outcome variables were analyzed using non-parametric testing described above due to non-normal distribution of the data. Pairwise Mann-Whitney U comparison tests were conducted between the HC group and all other 5 comparison groups (HC/BD × CU frequency comparison groups), as well as between CU frequency groups within the HC/BD cohorts. Significant (p < 0.05) interactions were reported, and the Bonferroni multiple comparisons correction was applied. All statistical analyses were performed using SPSS 28.0 (Chicago, IL, USA).

## Results

### No significant differences in CU patterns between healthy and BD participants

People with BD reported using cannabis to treat a greater number of symptoms (**Supplemental Data 1**; p < 0.05, d = 0.88). There was no significant difference in proportions of participants reporting recreational versus medicinal CU. There were no significant group differences in the reported weekly CU frequency.

### CU was associated with better decision-making in people with BD

**Fig. 1:**
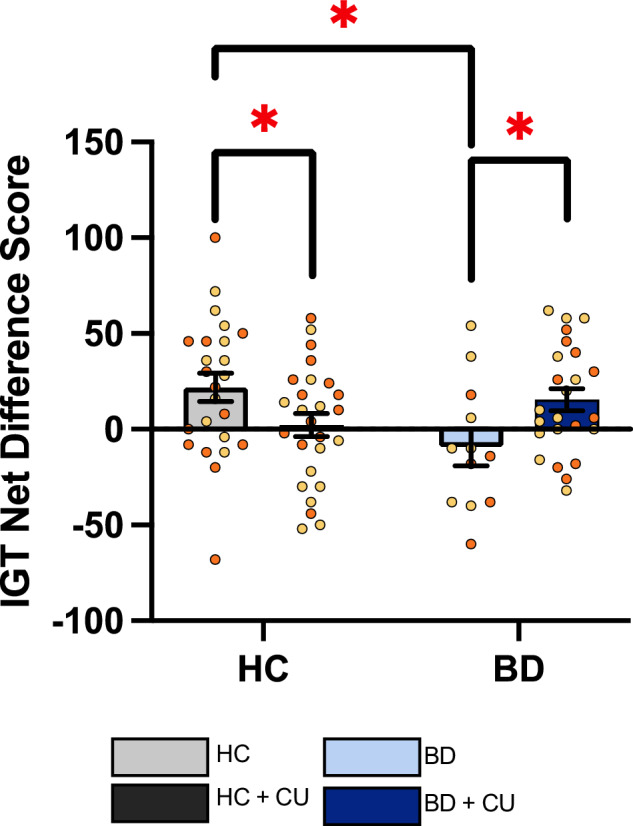
People with bipolar disorder (BD) that use cannabis (+CU) exhibited better decision-making in the Iowa Gambling Task (IGT) than non-CU BD participants. An overall interaction effect of BD × CU [F(1,82) = 9.39; p < 0.01; ${\eta }^{2}$ = 0.103] revealed that HC participants had significantly higher overall IGT scores compared to HC + CU and BD participants. BD + CU group had higher IGT scores than the BD group (p < 0.1) and, critically, did not differ from the HC group. *p < 0.05; Data presented as mean, SEM, and individual data points. Orange symbols represent males, yellow symbols represent females.

A significant BD × CU status interaction was observed on IGT Net Difference score (F(1,82) = 9.39; p < 0.01; ${\eta }^{2}$ = 0.103). IGT scores for the HC were higher than the HC + CU (t = −2.086, p = 0.042, d = −0.591) and BD (t = −2.50, p = 0.018, d = −0.883), but not the BD + CU group (Fig. 1; Supplementary Data 2a). The BD + CU group had higher IGT scores than the BD group (t = −2.328, p = 0.026, d = −0.823). Importantly, the BD + CU group exhibited comparable difference scores to HC group, indicating CU is not associated with worse decision-making in BD participants.

### BD and CU are associated with different decision making strategies

**Fig. 2:**
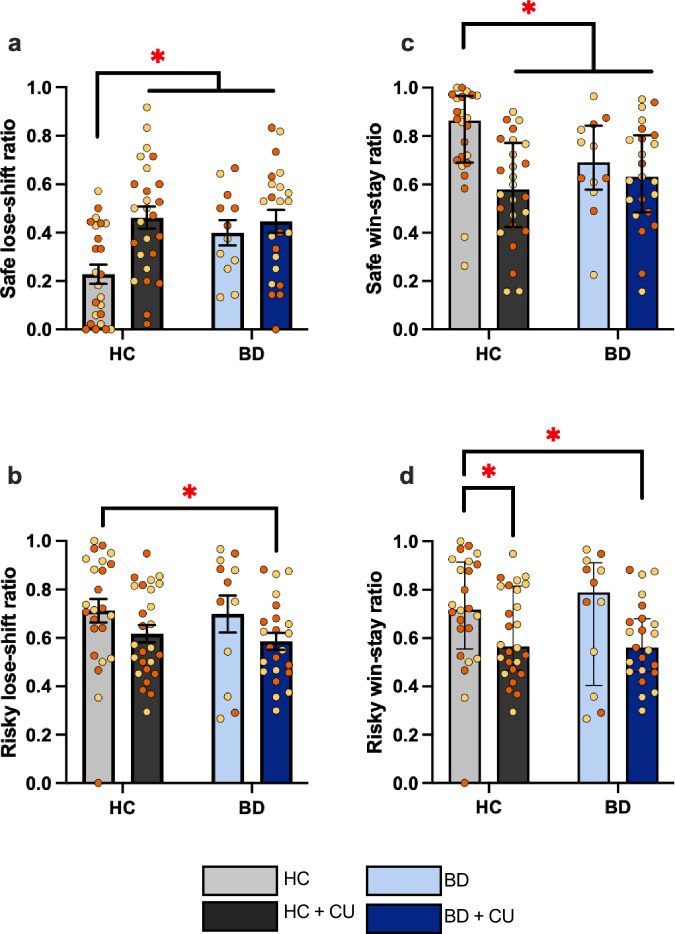
Bipolar disorder (BD) and cannabis use ( + CU) are associated with significantly higher safe lose-shift ratios and lower win-stay ratios (risky and safe) compared to non-cannabis using healthy comparison participants (HC). **a** HC participants had significantly lower safe lose-shift ratios compared to all other groups (p < 0.01). **b** The BD + CU group had lower risky lose-shift ratios compared to HC participants. *p < 0.05; **c** HC participants had significantly lower safe win-stay ratios compared to all other groups (ps <0.05). **d** HC participants had significantly higher risky win-stay ratios compared to CU groups (ps<0.05). *p < 0.05; Lose-shift data presented as mean, SEM. Win-stay data presented as median, interquartile range. Orange symbols represent males, yellow symbols represent females.

Replicating our prior observations [27], we observed that decision-making strategies were significantly different between BD and HC participants and extend these findings to BD and HC participants who use cannabis. There was no significant BD × CU interaction on safe lose-shift (SLS; F(1,81) = 3.68; p = 0.058; ${\eta }^{2}$ = 0.43), though a significant main effect of CU (F(1,81) = 8.36; p = 0.005; ${\eta }^{2}$ = 0.094, Fig. 2a) was detected. SLS was significantly lower in the HC group compared to all other groups (ps < 0.05, Supplementary Data 2b), indicating that both BD and CU were associated with a higher likelihood of switching from safe to risky choices following a loss. There was no BD × CU interaction on risky lose-shift (RLS), but there was a main effect of BD (F(1,80) = 4.03; p = 0.048; ${\eta }^{2}$ = 0.048, Fig. 2b). Visual inspection of the group differences indicated a higher RLS in the BD + CU participants compared to HC participants (Fig. 2b); thus, BD + CU participants were more likely to switch from a risky deck to a safe deck following a loss. HC participants also had significantly higher safe win-stay ratios (SWS; Fig. 2c) compared to all other groups (ps<0.05; Supplementary Data 2c). This difference indicates that both BD and CU was associated with a lower likelihood of repeatedly selecting from a safe deck following a reward. Risky win-stay ratios (RWS; Fig. 2d) were significantly lower in BD + CU and HC + CU participants relative to HC participants (ps<0.05, Supplementary Data 2b), indicating that CU is associated with a lower likelihood of continuing to select from a risky deck following a reward.

### Heavy, but not moderate, CU was associated with worse risk-based decision making

**Fig. 3:**
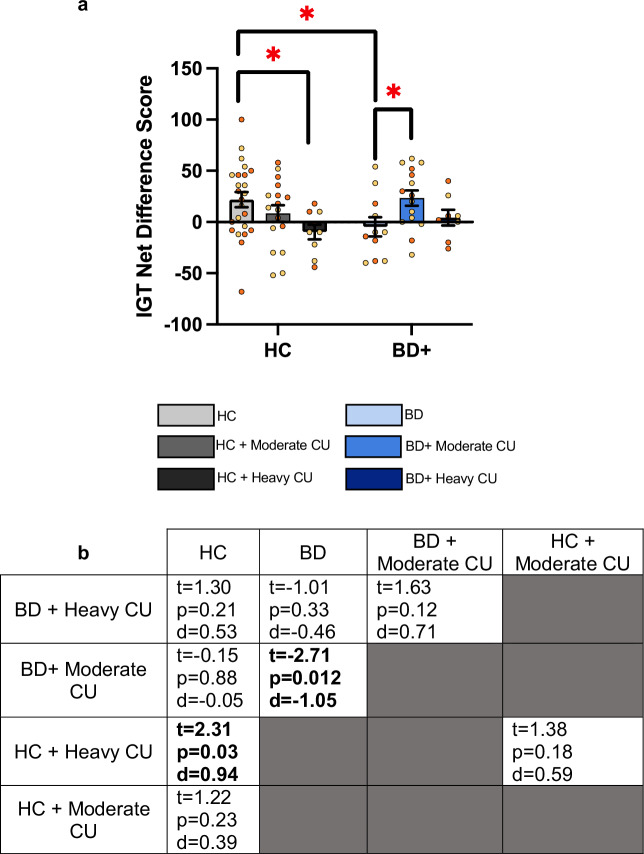
Moderate, but not heavy, cannabis use (CU) was associated with better decision-making in people with bipolar disorder (BD). **a** A significant BD status × CU frequency interaction was detected on total IGT net difference score (F(1,78) = 4.978; p = 0.009; effect size [${\eta }^{2}$ = 0.113]). Among the healthy comparison (HC) groups, both moderate and heavy CU was associated with worse IGT performance. The BD + moderate CU group, but not the BD + Heavy CU group, exhibited significantly higher IGT scores (safer decision-making) compared to the BD group. Data presented as median, interquartile range and individual data points. Orange symbols indicate males, yellow symbols indicate females. **b** Pairwise comparison test statistics for BD × CU frequency groups.

CU frequency has been associated with poor cognitive functioning in a dose-dependent manner in other populations [49, 50]. As such, we next sought to explore whether CU frequency was similarly associated with decision-making in people with BD. There was a significant BD status × CU frequency interaction detected on IGT net difference score (F(1,78) = 4.98; p = 0.009; effect size [${\eta }^{2}$ = 0.113], Fig. 3a). After applying the Bonferroni correction significance threshold (p < 0.006), there were no significant differences between BD × CU frequency groups. However, there were nominally significant (p < 0.05) differences. Within HC group, heavy CU was associated with worse IGT performance compared to no-CU participants (t = 2.31, p = 0.03, d = 0.94; Fig. 3b). Within the BD group, moderate CU was associated with better IGT performance compared to no CU (t = −2.71, p = 0.012, d = −1.05). There was no difference in IGT performance between the moderate CU (BD or HC) groups and the HC group. IGT secondary outcome variables were also analyzed by BD × CU frequency groups (Supplementary Data 3). Again, although no group differences met the Bonferroni correction significance threshold, there were some nominally significant differences in safe but not risky decision-making strategies. Notably, SWS was higher in the HC group compared to all other groups, except the BD + moderate CU group (Supplementary Data 3a).

### CU was associated with better functional medication management skills in BD

**Fig. 4:**
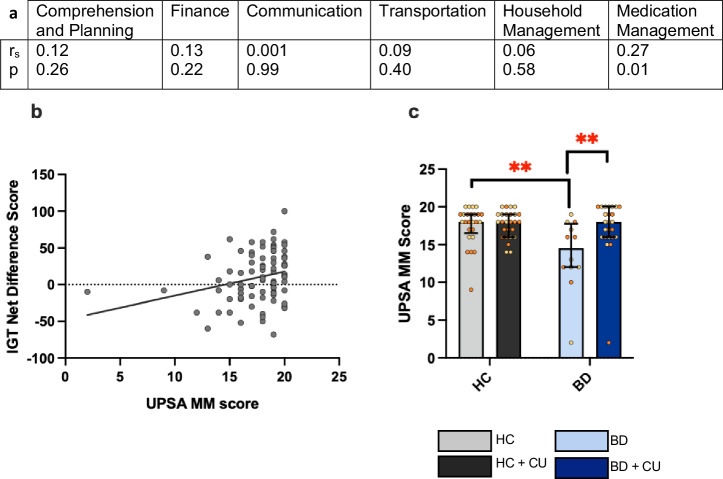
Non-cannabis using participants with bipolar disorder (BD) had lower UPSA Medication Management (MM) scores relative to BD participants who use cannabis ( + CU) and healthy comparison participants. **a** Correlations between UPSA sub-scores and IGT performance. **b** Net Iowa Gambling Task (IGT) score was positively correlated with UPSA MM score (r_s_=0.269, p < 0.05). **c** The BD participants had significantly lower UPSA MM scores compared to HC (p < 0.01) and BD + CU participants (p < 0.01). **p < 0.01; Data presented as median, interquartile range and individual data points. Orange symbols represent males, yellow symbols represent females.

There was no significant correlation between IGT performance and total UPSA score (r_s_ = 0.21, p = 0.054), however correlation analyses between UPSA sub-scores and IGT score (Fig. 4a) revealed that the UPSA Medication Management (MM) sub-score (r_s_ = 0.27, p < 0.05, Fig. 4b) was significantly correlated with IGT score. During the Medication Management section of the UPSA, participants are asked participants are asked to engage in a role play scenario during which they plan out a medication routine using 4 drugs taken over the course of one day with various restrictions (i.e., with or without meals, number of doses, etc.). Participants with higher IGT scores had better functional capability in the MM section of the UPSA-2. Nonparametric testing of UPSA total (H = 6.52, p = 0.10) and UPSA MM scores (H = 9.90, p = 0.019) revealed that median scores differed significantly between groups (Fig. 4c). Planned pairwise Mann-Whitney U tests revealed that BD participants had significantly lower median scores on the UPSA MM compared to all other groups (ps < 0.05, Supplementary Table 4). Consistent with IGT scores, BD + CU participants did not have significantly different UPSA MM scores compared to HC participants.

## Discussion

Here, we sought to determine whether chronic CU was associated with risk-based decision-making in people with BD. Consistent with our previous report, BD participants who did not use cannabis exhibited increased risk-preference in the IGT relative to HC participants [27]. Importantly, the current study demonstrated that decision-making by BD + CU participants was comparable to that of non-CU HC participants. Further, we also observed that CU was associated with reduced risk-preference (i.e., higher RLS) in BD but not HC participants. Similarly, only people with BD who do not use cannabis had significantly worse functional capacity compared to HC participants, specifically in the domain of medication management. Our secondary analyses suggest that the beneficial effects of CU on decision-making could be specific to moderate CU. Altogether, these data support the premise that people with BD may use cannabis because it confers cognitive benefits in this population.

A core neurochemical feature of BD mania is tonic hyperdopaminergia [51], likely mediated in-part by reduced DAT density [29] that may persist into euthymic and depressed states [28] (but see [52–55]). Studies of dopamine function in people with BD are few, although a heightened behavioral response to amphetamine has been reported relative to HC participants [56, 57], indicative of postsynaptic hyper-responsivity to dopamine [56]. PET imaging revealed that hyper-responsivity of the mesostriatal dopamine system (albeit presynaptic) was associated with risky decision-making in the IGT. Specifically, elevated amphetamine-induced increases in right ventrostriatal dopamine release were associated with poorer IGT performance in HC participants [58]. Additionally, in-task increases in striatal dopamine signaling predicted impaired IGT performance in individuals with gambling disorder, but better performance in HC participants [59]. The contribution of any *postsynaptic* dopamine hyper-responsivity to the IGT deficit of cannabis-abstinent people with BD remains to be directly evaluated, although its putative functional consequences could ostensibly be exacerbated by BD-associated DAT hypoexpression [28]. Indeed, prior studies observed elevated HVA levels in people with BD, and although these results were not stratified by cannabis use, they do support a hyperdopaminergic state present in people with BD.

While acute THC generally increases cortical and striatal dopamine release in humans [37, 60, 61] and animals [62–64], chronic exposure via regular CU leads to a long-term reduction in dopamine synthesis and transmission [65–67] (reviewed in [68, 69]:). Such a reduction could mitigate the BD-related elevations in dopamine signaling proposed above, possibly driving the improved IGT performance associated with moderate CU. This mechanism may also contribute to impaired performance of the HC + CU participants (both here and in previous reports [70–73]), given the association between IGT-related striatal dopamine release and better decision-making in healthy participants [59]. Interestingly, a positive association was previously also observed between plasma anandamide levels and IGT performance in healthy humans [74], strengthening the link between eCB signaling and decision-making. There is evidence to suggest a complex regulatory interaction between dopamine and anandamide [75–77]; thus, changes in anandamide levels in people with BD may occur in response to changes in dopamine transmission. Future studies should confirm the degree of involvement of pre- versus post-synaptic dopamine mechanisms on the effects of chronic CU in people with BD and their decision-making and increase sample sizes to evaluate the effects of chronic CU on anandamide.

IGT performance is predictive of clinical and functional outcome in BD. Emergence of hypomanic/manic symptoms has been predicted based on performance in a reward-based decision-making card task [78]. Previously, our group reported that poor cognitive task performance was correlated with worse functional capacity in people with BD patients, with manic/hypomanic patients performing significantly worse compared to depressed or euthymic BD patients [79]. Our current study supports previous findings that worse IGT performance was negatively correlated with higher levels of mania symptoms (Supplementary Data 5). Importantly, CU has previously been associated with worsening of mania and psychosis symptoms [80–82]. This association, combined with the high prevalence of CU in BD highlighted in the Introduction, has led to the hypothesis that CU may be a risk factor for the development of BD, rather than a form of self-medication. Although we do not have detailed data on whether CU was initiated prior to or after onset of BD symptoms, we did not observe a significant difference in mania symptoms between no-CU and CU groups (Supplementary Data 6). Though not statistically significant, BD + moderate CU participants reported lower mania symptoms compared to no CU and heavy CU. BD participants were clinically stable at the time of testing and sensitivity analyses conducted including Young Mania Rating Score and Hamilton Depression Rating Scale Score as a covariate did not alter the significance or directionality of the results. These data demonstrate that mania or depression severity may not be a primary driver of the differences in decision-making and are contrary to the hypothesis that CU contributes to worse BD mood symptoms. However, limiting our BD cohort to those without severe mood symptoms may also be a source of influence that should be explored in future studies. This research is particularly important, considering the altered neurochemical activity present during acute mood episodes in BD that may result in a differential response to cannabis.

In further support of the self-medication hypothesis of CU in BD is a recent study in which current CU in people with BD was associated with higher UPSA scores relative to BD participants who do not use cannabis [83]; our study replicates these findings by identifying an association between better functional capacity and chronic CU. With the caveat that we observed this finding with a single (albeit important and highly practical) domain of functional outcome (i.e., medication management; MM). Successful completion of the MM section of the UPSA requires grossly intact executive functioning and working memory; thus, it may be the case that CU in BD improves executive functions, such as decision-making, with beneficial effects on real-world functioning. Indeed, CU was associated with improved executive functioning in other populations likely required to manage extensive medication regimens [8, 48, 84], and the current data extends this premise to BD.

This study does have several limitations that require addressing however, particularly in that the cross-sectional design and static group comparisons limit any causal conclusions about causal CU effects on cognition. The IGT is also a laboratory-based measure which limits its reflection of decision-making across all contexts; nonetheless, the risk-based decision making assessed by the IGT was associated with real-world functional behaviors highly relevant to people with BD, such as substance use [85–87] and suicide risk [86, 88–90]. The CU frequency data make a strong case for direct effects of CU on cognition in people with BD; however, interpretation of the CU frequency findings are limited. For example, while the CU frequency as defined in the present study improves on prior literature in which weekly CU is loosely defined (e.g., 3–4/week may represent 3–4 days/week without considering # of times per day of use), these findings are not likely generalizable to other locations and participant populations where cannabis is not readily available and as such use frequency is inherently lower. Further, our dataset lacks critical data on potency and route of administration as most participants could not recall this information, which would have enhanced the interpretation of our results. Notably, participants who vape high cannabis concentrate 20 times a week may not be directly comparable to participants who smoke low-potency cannabis 4 times a week. Additionally, the primary reason for use (i.e., medicinal versus recreational) could also impact the THC potency individuals prefer to use; however, we did not observe a difference in cognitive performance between medicinal and recreational users. The differences in CU pattern variables highlights the need for future studies to consider: 1) standardized methods of reporting CU frequency; 2) collection of detailed CU variable data (e.g., route of administration and potency via certificate of analysis provided on cannabis products); and 3) use of multivariate or principal component analyses that include variables such as cumulative lifetime exposure (as suggested by Reis et. al [91]), potency, route of administration and CU frequency. Participants reported using products containing primarily THC or a combination of THC and CBD with additional constituents. Given the differential effects of CBD and THC on neurochemistry, any mechanistic interpretation of these findings is therefore limited. Nonetheless, the translational nature of the IGT enables future cross-species validation of this work, which could test the impact of different cannabinoids (e.g., THC or CBD), cannabis potency, and dosage/frequency of cannabinoid administration on IGT performance in the DAT knockdown mouse model of mania [18].

This study is also limited by a number of variables that may have independent effects on cognitive performance, such as age, mood symptoms, medication use, other substance or alcohol use and other sociodemographic variables. Sensitivity analyses suggest that neither age nor mood symptoms (mania and depression) have a significant effect on decision-making in this cohort. Reported medication use within the BD groups did not include detailed information such as dosage and frequency of medications, and as such could not be included as a covariate in our analyses. While we endeavored to collect dose and frequency information from the participants, many participants could not recall the exact prescribing details of their medications, not atypical for this population. We did not have access to their medical records. As shown in Table 1, there were no differences in the percentage of BD participants that reported medication use in any given category; however, future studies would benefit by including detailed medication use given the reported effects of some BD medications on cognitive functioning. Interestingly, a greater percentage of HC + CU participants reported current alcohol use compared to the other groups, though the number of drinks per week were below what might be considered problematic use (Table 1); future research should collect detailed other substance use and alcohol use to better understand how these factors may impact any potential cognitive effects of CU. Social determinants of health, such as income, housing, and social support, also differs in people with BD [92] or those with cannabis use disorder [93], relative to healthy adults, potentially influencing their decision-making processes and thereby contributing to the observed differences in risk-based decision making and functional capacity observed in our study. Finally, this study is limited by sample size, most notably in the non-CU BD group. Challenges in participant recruitment for this group were expected given the high prevalence of CU in people with BD.

**Fig. 5:**
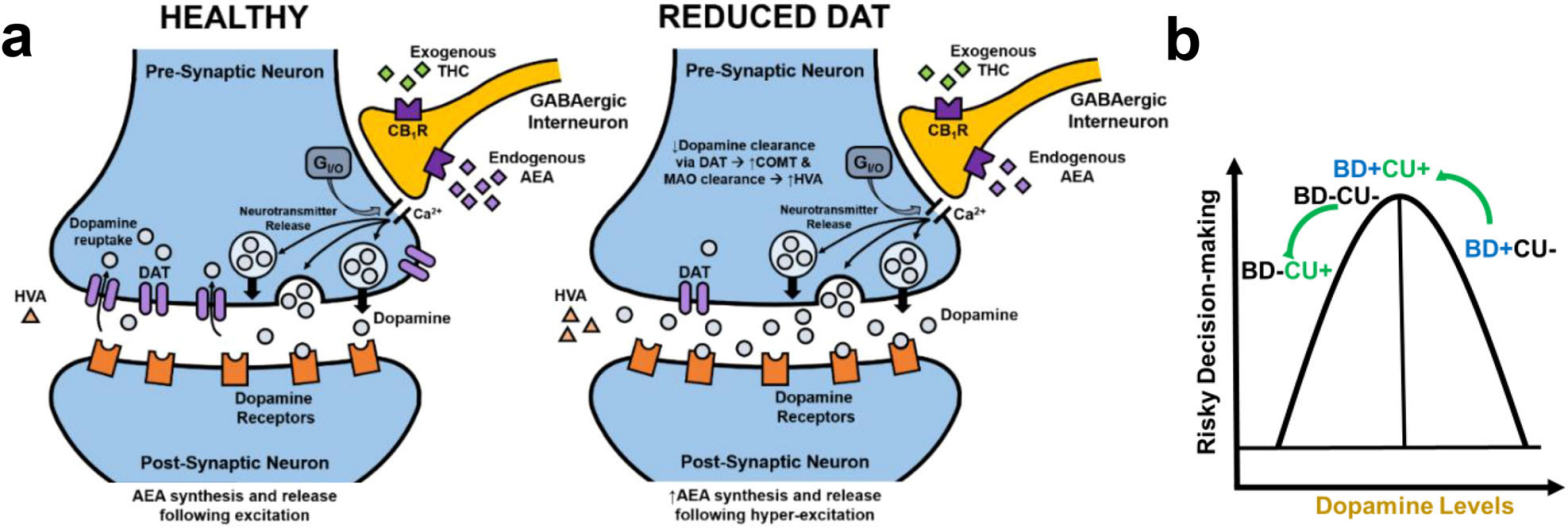
Proposed mechanism for the differential effects of chronic cannabis use (CU) in people with bipolar disorder (BD + ) versus healthy comparison participants (BD-). **a** Schematic illustrating interactions between dopaminergic and endocannabinergic neurotransmission in a healthy versus reduced-dopamine transporter (DAT) system. **b** Differential effects of CU on risky decision-making as measured by the Iowa Gambling Task (IGT) in BD + versus BD- individuals may reflect an inverted U-shaped relationship between dopamine levels and risk-based decision-making. Decision-making impairments in BD + non-CU participants are likely driven by elevated baseline dopamine levels, which may be normalized (reduced) by chronic CU. Meanwhile, CU may impair BD- decision-making by reducing dopamine tone to sub-optimal levels. CB1R: cannabinoid receptor 1; GABA: γ-aminobutyric acid; AEA: anandamide.

In summary, people with BD who use cannabis had decision-making and functional capacity comparable to non-CU HC participants. Based on the data presented here, our previous findings in mice with reduced DAT functioning, and preliminary evidence of elevated HVA levels in BD, we propose that elevated dopaminergic tone (HVA) may contribute to poorer performance in decision-making tasks in people with BD (Fig. 5a). Moreover, the association between better decision-making skills and CU in people with BD appears to be either frequency or potentially dose dependent, as BD participants reporting heavy CU exhibited similar IGT performance to the no-CU group, while only those reporting moderate CU performed better in the IGT (Fig. 5b). People with BD continue to report using cannabis for therapeutic purposes, including to remediate cognitive dysfunction. Thus, greater insight into the effects of cannabis on dopamine-eCB interactions would further elucidate putative treatments for BD. The limiting nature of cross-sectional studies such as these underscores the importance of clinical trials and the use of cross-species paradigms to determine causal effects of individual cannabinoids on eCB and dopamine neurotransmitter levels. Identification of treatment targets is critical considering the concerns of adverse effects of cannabis reported on other clinical outcomes, particularly mood and psychosis symptoms. Future studies may also endeavor to collect detailed cannabinoid content and potency information (e.g., through certificate of analysis) from cannabis products that are in current use by participants. Regardless the frequency or potentially dose-dependent effects on decision-making strategies and functional capacity reported here, and the negligible differences in mood symptom severity in the BD groups, suggest that CU practices could be appropriately managed in people with BD to improve cognitive function, a possibility that merits further exploration.

## Supplementary information

Supplemental material

## Data Availability

All data produced in this study is shown in manuscript and supplementary information, and unprocessed data are available from the corresponding author on reasonable request.
