# Supplementary material for: Chronic cannabis use in people with bipolar disorder is associated with comparable decision-making and functional outcome to healthy participants
Source: Transl Psychiatry. 2025 Nov 27;15:506. doi: 10.1038/s41398-025-03718-4 (PMC12660854; doi:10.1038/s41398-025-03718-4)
Supplement: Supplementary file 1 — Supplemental material [file 41398_2025_3718_MOESM1_ESM.docx]

**Supplemental Data**

|  | **HC**  **(n=26)** | **BD**  **(n=25)** | **Group differences** |
| --- | --- | --- | --- |
| **Average cumulative weekly use** | 20.7(13.9) | 26.3(23.1) | ns; t =1.2 |
| **Recreational vs. medical use** | Mostly Recreational (44%)  Mostly Medicinal (20%)  Both (36%) | Mostly Recreational (39%)  Mostly Medicinal (28%)  Both (32%) | ns; χ^2^=5.0 |
| **# symptoms** | 3(3.4) | 7(4.2) | p<0.01; t =3.1 |
| **Top 3 common reasons/symptoms for medical use** | Anxiety (52%)  Sleep (48%)  Stress (48%) | Anxiety (75%)  Sleep (53%)  Depression (61%) |  |

**Supplemental Data 1: Cannabis use patterns and factors in healthy comparison (HC) and bipolar participants (BD).** Data presented as mean(standard deviation), % within HC or BD, and counts.

| **a** | HC | BD |
| --- | --- | --- |
| BD + CU | t=0.69  p=0.50  d=0.20 | **t=2.33**  **p=0.026**  **d=0.82** |
| BD | **t=2.50**  **p=0.018**  **d=0.88** |  |
| HC+CU | **t=2.09**  **p=0.042**  **d=0.59** |  |

| **c** |  | HC | BD |
| --- | --- | --- | --- |
| **Safe win-stay** | BD + CU | **U=146**  **z=-2.77**  **p=0.006** | U=120  z=-0.63  p=0.53 |
|  | BD | **U=83**  **z=-2.03**  **p=0.042** |  |
|  | HC+CU | **U=155**  **z=-3.05**  **p=0.002** |  |
| **Risky win-stay** | BD + CU | **U=130.5**  **z=-2.94**  **p=0.003** | U=92  z=-1.6  p=0.11 |
|  | BD | U=134  z=-0.14  p=0.89 |  |
|  | HC+CU | **U=187.5**  **z=-2.23**  **p=0.025** |  |

| **b** |  | HC | BD |
| --- | --- | --- | --- |
| **Safe lose-shift** | BD + CU | **t=3.56**  **p<0.001**  **d=1.04** | t=-0.62  p=0.54  d=-0.22 |
|  | BD | **t=2.57**  **p=0.015**  **d=0.91** |  |
|  | HC+CU | **t=3.86**  **p<0.001**  **d=1.09** |  |
| **Risky lose-shift** | BD + CU | **t=2.33**  **p=0.025**  **d=0.69** | t=-1.16  p=0.26  d=-0.41 |
|  | BD | t=0.72  p=0.48  d=0.26 |  |
|  | HC+CU | t=0.22  p=0.83  d=0.063 |  |

**Supplementary Data 2**: Pairwise comparison test statistics for IGT outcome variables. **A)** T-test statistics for IGT Net Difference Score group differences **B)** T-test statistics for lose-shift ratio group differences and **C)** Mann-Whitney U test statistics for win-stay ratios group differences.

|  |  | HC/CU- | BD/CU- |
| --- | --- | --- | --- |
| **Safe lose-shift** | BD/CU+ | **t=3.56**  **p<0.001**  **d=1.04** | t=-0.62  p=0.54  d=-0.22 |
|  | BD/CU- | **t=2.57**  **p=0.015**  **d=0.91** |  |
|  | HC/CU+ | **t=3.86**  **p<0.001**  **d=1.09** |  |
| **Risky lose-shift** | BD/CU+ | **t=2.33**  **p=0.025**  **d=0.69** | t=-1.16  p=0.26  d=-0.41 |
|  | BD/CU- | t=0.72  p=0.48  d=0.26 |  |
|  | HC/CU+ | t=0.22  p=0.83  d=0.063 |  |

**a**

**Supplemental Data 3: Decision making strategies in people with bipolar disorder (BD) and healthy comparison participants by cannabis use (CU) frequency. A)** HC participants had nominally higher safe win-stay ratios compared to all other groups, except the BD + moderate CU group. BD + Heavy CU group also had nominally lower safe win-stay ratios compared to the BD group. **B)** HC participants had nominally lower safe lose-shift ratios compared to all other groups. **C)** There were no differences in risky win-stay ratios between HC/BD and CU frequency groups. **D**) There were no differences in risky lose-shift ratios between HC/BD and CU frequency groups. Group comparisons were conducted through pairwise Mann-Whitney U tests; *****p<0.05 prior to Bonferroni correction. Data presented as median ± interquartile range and individual data points. Orange symbols indicate males, yellow symbols indicate females.

|  | HC/CU- | BD/CU- |
| --- | --- | --- |
| BD/CU+ | U=267  z=-0.19  p=0.85 | **U=63**  **z=-2.62**  **p=0.009** |
| BD/CU- | **U=57.5**  **z=-2.92**  **p=0.003** |  |
| HC/CU+ | U=282  z=-0.59  p=0.56 |  |

**Supplemental Table 4:** Pairwise comparison test statistics for UPSA medication management sub-score.


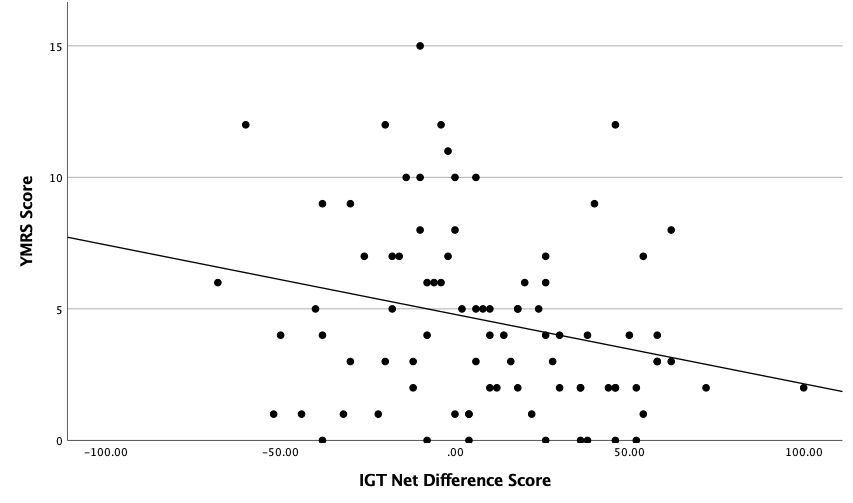


**Supplemental Data 5:** Young Mania Rating Scale (YMRS) Score is negatively correlated with Iowa Gambling Task (IGT) Score (r_s_=-0.252, p=0.019).

|  | **HC+ Moderate CU (n=17)** | **HC+ Heavy CU (n=8)** | **BD** | **BD+ Moderate CU (n=16)** | **BD+ Heavy CU (n=8)** | **Group differences** |
| --- | --- | --- | --- | --- | --- | --- |
| Age | 27.8 (6.7) | 35 (8.9) |  | 27.6 (8.5) | 31.8 (8.8) | ns |
| Average cumulative weekly use | 13.1(5.9) | 36.8(12.3) |  | 14(7) | 50.8(24.7) | ns between HC and BD |
| Age of CU onset | 20.5(3.6) | 21.9(10.7) |  | 22(11.7) | 28(9.9) | ns between HC and BD |
| % currently medicated |  |  | 91% | 71.4% | 75% | ns |
| HAMD |  |  | 9.3(5.4) | 6.9 (3.9) | 9.9(6.0) | ns |
| YMRS |  |  | 6.7(4.5) | 4.4(2.9) | 7.6(2.8) | ns |

**Supplementary Data 6:** Clinical and cannabis use characteristics between non, moderate and heavy cannabis users. Abbreviations; healthy comparison participants (BD-), participants with BD (BD+), cannabis use (CU), Hamilton Depression Rating Scale (HAMD), Young Mania Rating Scale (YMRS). Data presented as means (standard deviation) unless otherwise indicated.
